# Commemorating the Landmark Advances in Our Understanding of Mucormycosis by Dimitrios P. Kontoyiannis

**DOI:** 10.3390/jof11060435

**Published:** 2025-06-06

**Authors:** Thomas J. Walsh

**Affiliations:** 1 Center for Innovative Therapeutics and Diagnostics, Richmond, VA 23220, USA; tjwalsh@citdx.org; 2 Departments of Medicine and Microbiology & Immunology, University of Maryland School of Medicine, Baltimore, MD 21201, USA

**Keywords:** Dimitrios P. Kontoyiannis, mucormycosis, epidemiology, pathogenesis, diagnosis, antifungal therapy

## Abstract

Writing with the perspective of a mentor, friend, and colleague, I am elated to contribute to this commemorative issue of the *Journal of Fungi*, which is dedicated to the landmark contributions of Dr. Dimitrios P. Kontoyiannis, who has greatly advanced our understanding of the epidemiology, pathogenesis, diagnosis, and treatment of mucormycosis. Through his efforts and leadership, the four pillars in the management of mucormycosis have been made much stronger: early diagnosis, the rapid initiation of antifungal therapy, augmentation of the host’s defenses, and surgical intervention.

## 1. His Training and Education as a Physician–Scientist

Training to be and developing and succeeding as a physician–scientist in the field of medical mycology is fraught with formidable challenges. At the same time, the challenges presented by life-threatening invasive fungal diseases in our patients are also daunting threats. Among these invasive fungal diseases, mucormycosis ranks among the most lethal and destructive infections. Dimitrios has tenaciously met both challenges in his role as an esteemed physician–scientist who has made groundbreaking contributions to our understanding of the pathogenesis, diagnosis, and treatment of mucormycosis.

As physician–scientists, we strive to impart three special qualities at our patients’ bedsides: science, compassion, and wisdom. Dr. Dimitrios Kontoyiannis epitomizes all three esteemed attributes.

**Figure 1:**
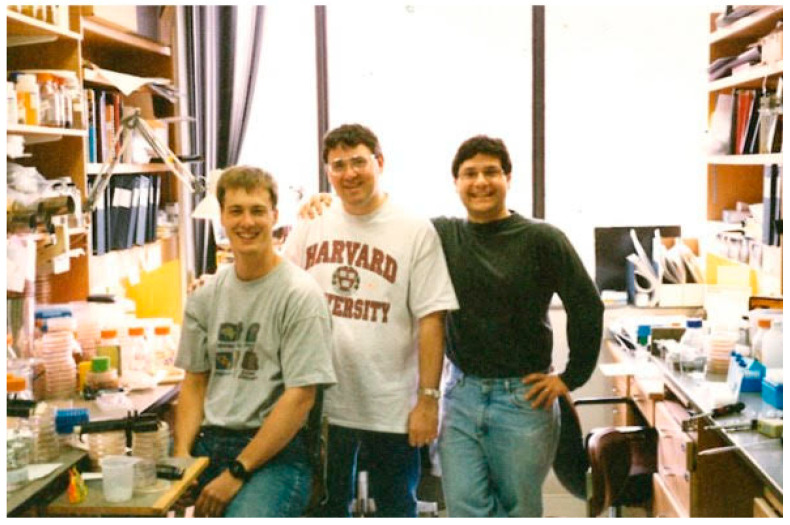
Dimitrios Kontoyiannis as a Harvard–MIT Clinical Investigators Fellow at the Whitehead Institute for Biomedical Sciences/MIT.

While some physician–scientists may excel in selected areas of the epidemiology, pathogenesis, diagnosis, and treatment of a given disease, Dimitrios’s training and dedication have enabled him to pursue all of these facets for mucormycosis and more profoundly understand and manage this life-threatening disease. Graduating as valedictorian *summa cum laude* from the National and Kapodistrian University of Athens Medical School in Greece, Dimitrios was already distinguished at an early stage of his career as a leading medical scholar. He subsequently served as Chief Resident during his training in Internal Medicine at Baylor College of Medicine in Houston, Texas. Dimitrios established himself as a pre-eminent physician–scientist in infectious diseases through Fellowships in Infectious Diseases at the Massachusetts General Hospital, his master’s degree in Clinical Sciences from Harvard Medical School, and a Harvard/Massachusetts Institute of Technology (MIT) Clinical Investigators Fellowship at the Whitehead Institute for Biomedical Sciences/MIT (Figure 1).

With increasing interest in infections in immunocompromised patients, Dimitrios was recruited as a member of the junior faculty at the MD Anderson Cancer Center in Houston, Texas, where he would embark upon a career lasting more than three decades, making major contributions to our understanding of mucormycosis. Compelled by the devastating morbidity and tragic mortality due to mucormycosis that he witnessed at patients’ bedsides, Dimitrios, in the spirit of a dedicated physician–scientist, asked the critical questions of what the burden of mucormycosis was, why these infections were so lethal, and how their outcomes could be improved.

## 2. Epidemiology

Dimitrios’s studies of its epidemiology, risk factors, and prognostic indicators have increased the general clinical awareness and bedside understanding of mucormycosis [1,2,3,4,5,6,7,8,9,10,11,12]. His initial study of 24 patients with hematologic malignancies at MDACC in the 1990s found that favorable outcomes were correlated with surgical interventions, the absence of pulmonary infections, recovery from neutropenia, and a cumulative dose of 2000 mg of liposomal amphotericin B. Dissemination was observed in 58% of patients in whom postmortem examinations were performed. The sensitivity of cultures to detect pulmonary mucormycosis was low. Mortality rates were high (67%). He and his colleagues concluded that early treatment with high-dose amphotericin B, the use of aggressive surgery, and immune reconstitution could increase the probability of survival in this vulnerable patient population [2].

Building upon his parallel experiences in managing invasive pulmonary aspergillosis, Dimitrios and his colleagues studied the predictors of pulmonary zygomycosis versus those for invasive pulmonary aspergillosis in patients with cancer. This seminal study found that concomitant sinusitis, voriconazole administration, multiple (≥10) pulmonary nodules, and pleural effusion were independent predictors of mucormycosis [3]. High APACHE II scores, severe lymphocytopenia (<100/mm^3^), and elevated serum levels of LDH at the time of a PM diagnosis were independent markers of rapid disease progression and death. When baseline APACHE II scores were incorporated into the model, a weighted risk score of >22 was associated with 8-fold higher rates of mortality (*p* < 0.0001) [4].

In collaboration with the Mycoses Study Group and the Centers for Disease Control (CDC), Dimitrios guided the data analysis and reporting for 23 transplant centers in the Transplant-Associated Infection Surveillance Network from 2001 to 2006 to study 169 non-Aspergillus mold infections, including 105 cases of mucormycosis [5]. The 12-month cumulative incidence of mucormycosis was 0.29% for hematopoietic cell transplant (HCT) recipients and 0.07% for solid organ (SOT) recipients. Moreover, the incidence of mucormycosis was found to increase during the study period. Contemporaneously, this was the largest multicenter study of non-Aspergillus mold infections among transplant recipients to have been conducted.

Further elucidating the national impact of mucormycosis in the United States, Dimitrios and his colleagues found a prevalence of mucormycosis-related hospitalization during January 2005–June 2014 of 1.2/100,000 discharges, with a median length of stay of 17 days, a mortality rate of 23%, a readmission rate of 37%, and a mean cost of hospitalization of USD 112,419 [6]. This report was the first combined nationwide epidemiological and fiscal analysis to show the destructive morbidity and financial burden of mucormycosis.

Dimitrios and his staff were among the first to collectively and comprehensively review the epidemiology, clinical characteristics, and treatment of the less common genera of Mucormycetes [7]. While participating in the overall mission of the International Osteoarticular Mycoses Working Group [8], Dimitrios also contributed to the first comprehensive literature review of osteoarticular mucormycosis [9].

Dimitrios and his colleagues were at the epidemiological forefront of understanding the global surge in cases of mucormycosis during the COVID-19 pandemic. However, during its outbreak, neither the epidemiological factors nor the clinical manifestations and outcomes of COVID-19-associated mucormycosis (CAM) were well described.

In response to this gap in our knowledge, Dimitrios and his colleagues reviewed the 41 cases of COVID-19-associated mucormycosis (CAM) that had been published and described the convergence of two “storms” of DM and COVID-19 resulting in this devastating mycotic complication [10]. These patients most frequently presented with rhino-orbital and rhino-orbital-cerebral mucormycosis. Diabetes mellitus (DM) constituted the major apparent risk factor in 94% of cases, especially in those with poorly controlled DM (67%) and severe or critical COVID-19 (95%).

Continuing to interrogate the question of the disproportionately high incidence of CAM in India in comparison to that in other countries, Dimitrios and his colleagues found a critical intersection between host and environmental factors that likely contributed to the emergence of CAM [11]. They reviewed the medical records for all patients diagnosed with biopsy-proven CAM and appropriate control patients from seven hospitals in New Delhi and the National Capital Region. Multivariate logistic regression models identified newly diagnosed diabetes mellitus, active cancer, and severe COVID-19 infections as independent risk factors. Increased environmental spore concentrations in the weeks preceding the peak incidence of CAM correlated with increased temperatures, elevated evaporation, and decreased relative humidity, all of which will have facilitated sporangiospore propagation, transmission, and acquisition. He and his colleagues further hypothesized that sporangiospores may have been present in high concentrations in enclosed shelters where animal dung and other organic matter were burnt for fuel by highly vulnerable patients with DM [12]. Thus, Dimitrios again emerges as a thoughtful pathophysiological, microbiological, and environmental analyst elucidating challenging and unprecedented cases of CAM’s emergence.

## 3. Pathogenesis

Dimitrios has contributed extensively to our understanding of the pathogenesis of mucormycosis [13,14,15,16,17,18,19,20,21,22,23,24,25,26,27,28,29]. His early studies characterized the increased virulence of Cunninghamella bertholletiae [13,17], angioinvasion [14], the effect of pre-exposure to triazoles on *Rhizopus* virulence [19], and the effect of glucocorticosteroids on hyphal growth [20]. As iron acquisition is a critical factor in the virulence of mucormycosis, Dimitrios and his colleagues investigated the impact of iron deprivation in inducing apoptosis in *Rhizopus oryzae* in vitro [21], and in collaboration with his mentee, Dr George Chamilos, found that intracellular macrophage iron restriction was an important regulatory mechanism of the pulmonary host defense against *Rhizopus* spp. [24]. Dimitrios’s collaboration with Ashraf Ibrahim’s laboratory led to the key discovery of the impact of bicarbonate correction of metabolic acidosis on host defenses and the virulence of experimental mucormycosis [22].

Dimitrios studied the pathogenesis of mucormycosis in a series of complementary innovative model systems [25]. He introduced the first *Drosophila melanogaster* fly model [18] and an immunosuppressed zebrafish model of mucormycosis [23]. The fly model of mucormycosis has been used widely to elucidate the virulence mechanisms and host–pathogen interactions for mucormycosis.

In the wake of several catastrophic tornado and tsunami events leading to increased cases of musculoskeletal mucormycosis, Dimitrios and his colleagues spearheaded a cutting-edge study aiming to characterize the effect of tornadic shear stress on the development of a calcineurin-dependent hypervirulent phenotype of Mucorales pathogens in the aforementioned fly model [26]. In addressing trauma-associated mucormycosis in greater depth, Dimitrios and his colleagues hypothesized that immune paralysis may play a critical role in increasing the susceptibility to locally invasive musculoskeletal mucormycosis [27].

## 4. Diagnosis

Emphasizing the importance of the early diagnosis and initiation of treatment in mucormycosis, Dimitrios has contributed greatly to this critical pillar of management [30,31,32,33,34,35,36,37,38,39,40,41]. Under the leadership of Dimitrios, Chamilos and colleagues conducted a landmark study that documented how the early initiation of antifungal therapy could nearly double patients’ survival [30].

Paramount to the early detection of pulmonary and disseminated mucormycosis are diagnostic imaging tools [31,32,33,34,35]. Dimitrios greatly advanced our understanding of computed tomography (CT) imaging of pulmonary mucormycosis, especially in characterizing the diagnostic significance of the reverse halo sign [32,33].

Dimitrios and his team have also advanced the development of molecular diagnostic systems for the detection of Mucorales in the bronchoalveolar lavage, plasma, whole blood, and tissues [35,36,37,38,39,40]. These systems represent important cornerstones in the molecular detection of mucormycosis.

## 5. Treatment

Dimitrios has made major contributions to our understanding of the treatment of mucormycosis [42,43,44,45,46,47,48,49,50,51,52,53,54,55,56,57,58,59,60,61,62,63,64,65,66,67,68,69,70,71,72,73,74]. Recognizing the limitations of the prevailing treatment strategies in use up to 2012 [43,44,45], Dimitrios, his colleague Russel Lewis, and their laboratory staff embarked on a series of preclinical studies of the in vitro and in vivo pharmacodynamics of single-agent antifungal agents, including an amphotericin B lipid complex, liposomal amphotericin B, caspofungin, posaconazole, and isavuconazole [46,47,48,49,50,51]. With the additional aim of augmenting the antifungal activity of posaconazole, he and his team investigated several therapeutic strategies utilizing novel mechanisms, including tacrolimus, hyperthermia, and mitochondrial pathway inhibition, in murine models of pulmonary mucormycosis [52,53,54]. This team further explored additional novel therapeutics, including deferasirox, antimicrobial peptidomimetics, HMG-CoA reductase inhibitors (statins), hyperbaric oxygen, and colistin [55,56,57,58,59].

With expansions in the use of mold-active triazoles for antifungal prophylaxis in high-risk patients suffering from hematological malignancies, Dimitrios investigated the problem of breakthrough infections caused by Mucorales and identified an enhanced virulent phenotype of *Rhizopus oryzae* [60,61,62,63,64]. With the advent of echinocandins and isavuconazole, he and his team also studied combination antifungal therapy [65] and concluded in a rigorous propensity score analysis that initial combination therapy had no effect on the outcomes in patients with mucormycosis and hematological malignancies [66].

As an internationally distinguished leader in the management of mucormycosis and other invasive fungal diseases, Dimitrios continues to guide the development of the therapeutic recommendations for mucormycosis [67,68,69]. These publications include guidelines for the treatment of mucormycosis, the length of therapy, and antifungal stewardship.

Throughout his career, Dimitrios has endeavored brilliantly to develop innovative approaches to the treatment of mucormycosis, including more recently introduced investigational antifungal agents, PD-1/PD-L1 inhibitors, other checkpoint inhibitors, and activated T-cells, and to understanding the effects of climate change [70,71,72,73,74], with the ultimate objective of saving lives and improving outcomes in our most vulnerable patients at risk of and afflicted with mucormycosis.

**Figure 2:**
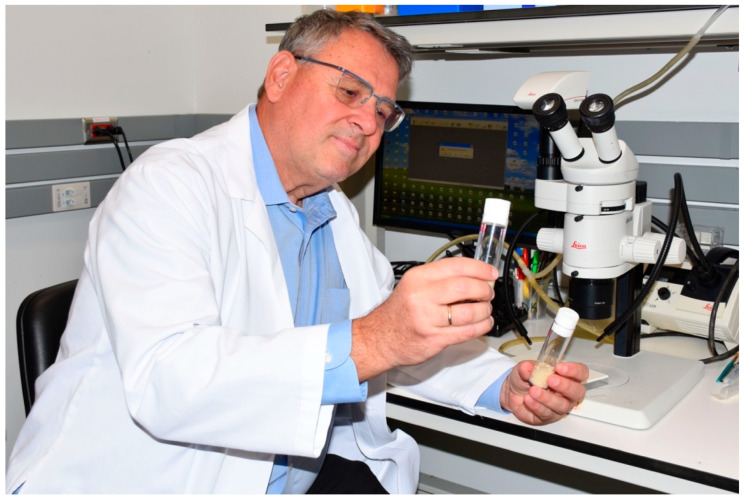
Dimitrios P. Kontoyiannis, MD, ScD, PhD (Hon), FIDSA, FAAM, FAAAS, AAP, Robert C Hickey Chair in Clinical Care, Deputy Head, Division of Internal Medicine, President Mycoses Study Group Education and Research Consortium, ECMM Diamond Excellence in Mycology Center.

Finally, while Dimitrios has received many well-deserved accolades, honors, and awards for his impactful and sustained research achievements, it is also worth pointing out that he remains a passionate master clinician, a devoted mentor to a generation of talented young mycologists, and a formidable role model of excellence and scholarship. These attributes underlie his stellar influence in modern mycology and infectious diseases (Figure 2).
